# Frequent Intra-Subtype Recombination among HIV-1 Circulating in Tanzania

**DOI:** 10.1371/journal.pone.0071131

**Published:** 2013-08-05

**Authors:** Ireen E. Kiwelu, Vladimir Novitsky, Lauren Margolin, Jeannie Baca, Rachel Manongi, Noel Sam, John Shao, Mary F. McLane, Saidi H. Kapiga, M. Essex

**Affiliations:** 1 Kilimanjaro Christian Medical Centre and College, Tumaini University, Moshi, Tanzania; 2 Department of Immunology and Infectious Diseases, Harvard School of Public Health, Boston, Massachusetts, United States of America; 3 London School of Hygiene and Tropical Medicine, London, United Kingdom; 4 Kilimanjaro Reproductive Health Program, Moshi, Tanzania; Lady Davis Institute for Medical Research, Canada

## Abstract

The study estimated the prevalence of HIV-1 intra-subtype recombinant variants among female bar and hotel workers in Tanzania. While intra-subtype recombination occurs in HIV-1, it is generally underestimated. HIV-1 *env* gp120 V1-C5 quasispecies from 45 subjects were generated by single-genome amplification and sequencing (median (IQR) of 38 (28–50) sequences per subject). Recombination analysis was performed using seven methods implemented within the recombination detection program version 3, RDP3. HIV-1 sequences were considered recombinant if recombination signals were detected by at least three methods with p-values of ≤0.05 after Bonferroni correction for multiple comparisons. HIV-1 in 38 (84%) subjects showed evidence for intra-subtype recombination including 22 with HIV-1 subtype A1, 13 with HIV-1 subtype C, and 3 with HIV-1 subtype D. The distribution of intra-patient recombination breakpoints suggested ongoing recombination and showed selective enrichment of recombinant variants in 23 (60%) subjects. The number of subjects with evidence of intra-subtype recombination increased from 29 (69%) to 36 (82%) over one year of follow-up, although the increase did not reach statistical significance. Adjustment for intra-subtype recombination is important for the analysis of multiplicity of HIV infection. This is the first report of high prevalence of intra-subtype recombination in the HIV/AIDS epidemic in Tanzania, a region where multiple HIV-1 subtypes co-circulate. HIV-1 intra-subtype recombination increases viral diversity and presents additional challenges for HIV-1 vaccine design.

## Introduction

Recombination plays an important role in the evolution of retroviruses and contributes to HIV-1 diversity [1]. Recombinant viruses can be generated when two distinct viruses infect a single cell, either simultaneously, by a single transmission of multiple viral variants, or sequentially, in multiple transmission events [2]. HIV-1, like other retroviruses, contains two copies of RNA per virion that encode the HIV-1 genome. When a cell becomes infected by two RNAs with sequence differences, the RNA genomes can be co-packaged and transferred to viral progeny. In the presence of two distinct viral RNA templates in the cell, the viral reverse transcriptase during reverse transcription can switch from one RNA template to another, generating a mosaic HIV genome containing genetic information from both RNAs [1].

Recombination can occur between viruses of the same HIV-1 subtype, known as intra-subtype recombination, or between viruses belonging to different HIV-1 subtypes, known as inter-subtype recombination [2], [3]. Several studies reported that recombination occurs between HIV-1 group M subtypes (inter-subtype recombination) [1], [4], HIV-1 groups M and O (inter-group recombination) [5], as well as within subtypes of HIV-1 group M (intra-subtype recombination) [3], [6]. To date, most of the previous studies have focused on HIV-1 inter-subtype recombination [1], [7]–[9], while only a few have addressed the HIV-1 intra-subtype recombination in subtype B [3], [6], [10]–[17], and subtype C [18].

Recently we reported prevalence and distribution of HIV-1 subtypes and inter-subtype recombinant viruses among female bar and hotel workers in Moshi, Tanzania [19]. The proportion of circulating inter-subtype recombinants in this population was relatively low at 8%, but the prevalence of HIV-1 infections with multiple variants was found to be 27% [19]. The presence of multiple viral variants can facilitate HIV-1 recombination including recombination between viruses of the same subtype.

The clinical and biological relevance of HIV-1 intra-subtype recombination remains poorly understood. Previous studies have shown that inter-subtype recombination can alter cell tropism, viral pathogenicity, antiretroviral drug susceptibility, the diagnostic accuracy of serologic and molecular assays, as well as disease progression [2], [20]–[26]. Viral recombination can also distort the phylogenetic signals which can alter the accuracy of analysis [18], [27], [28]. HIV-1 inter-subtype recombination can be detected relatively easily [29]. In contrast, HIV-1 intra-subtype recombination has been difficult to detect and has therefore been commonly understudied [18].

Historically, for identification of inter-subtype recombinants of HIV-1, subtype reference sequences have been required. However, due to sequence similarity and lack of reference sequences, routine identification of HIV-1 intra-subtype recombinants was limited to cases with known sequences of transmitted multiple viral variants that could be used as references for recombination analysis. The introduction of recombination detection software RDP3 [30] combined with single-genome amplification and sequencing (SGA/S) allowed us to estimate the frequency of intra-subtype recombination in a cohort of female bar and hotel workers in Moshi, Kilimanjaro region, Tanzania.

## Methods

### Ethics Statement

This study was conducted according to the principles expressed in the Declaration of Helsinki, and was approved by the institutional review boards at the Kilimanjaro Christian Medical Centre (KCMC), Tanzania National Institute for Medical of Research, and Harvard School of Public Health (HSPH). All study subjects provided written informed consent for participation in the study.

### Study Subjects

Briefly, 800 women employed in the hotels, bars and guest houses in Moshi town of northern Tanzania were enrolled in the prospective cohort study between December 2004 to March 2007 [19], [31], [32]. Assessment of HIV-1 status, recruitment of study subjects, description of the cohort, and sampling procedures have been described elsewhere [19], [31], [32]. Subjects were followed-up quarterly over one year. At each study visit women were examined, interviewed about their sexual behavior and HIV-related risk factors, and blood samples were collected.

Among 800 subjects, 139 (17%) were HIV-1 positive by serological testing [19], [31], [32]. A subset of 50 out of 139 HIV-1 positive subjects with at least two samples collected one year apart has been characterized recently [19].

A total of 45 out of 50 subjects infected with non-recombinant HIV-1 subtypes A, C, or D [19] were selected for intra-subtype recombination analysis in this study. Five subjects were excluded from analysis: four were infected with HIV-1 inter-subtype recombinant viruses, and one subject was infected with HIV-1 subtype A1 but only one viral sequence was available. The median age of the 45 subjects at the study entry was 30 years (IQR 26–37).

HIV-1 RNA in plasma and mean pairwise genetic distances were assessed at two time points over one year [19].

### Single Genome Amplification and Sequencing (SGA/S)

Isolation of genomic DNA from peripheral blood mononuclear cell separation (PBMC) using QIAamp DNA Mini Kit and HIV-1 RNA isolation from plasma using QIAamp viral RNA Mini kit (QIAGEN, Valencia, CA) have been described previously [19]. A fragment of HIV-1 *env* gp120 spanning the V1-C5 region (nucleotide position 6,615–7,757; HXB2 numbering at [4] was amplified using a modified SGA/S technique [33], [34] based on the limiting dilution [35]. Purified amplicons were directly sequenced on both strands on the ABI 3730 DNA analyzer using the BigDye technology.

### Phylogenetic Analysis and HIV-1 Subtyping

The HIV-1 *env* quasispecies were classified into HIV-1 subtypes as described previously (Kiwelu et al., 2012). The recombination identification program (RIP 3.0; [4] and REGA HIV-1 subtyping tool [36] were used to screen for evidence of HIV-1 inter-subtype recombination [19]. The breakpoints in inter-subtype recombinant quasispecies were detected by bootscanning analysis using SimPlot v3.5.1 [37]. A subset of 1795 HIV-1 *env* quasispecies from 45 subjects infected with HIV-1 subtypes A1, C or D were identified as non-recombinant based on inter-subtype categorization.

The sequences described in this paper were submitted to the GenBank and are available under the following accession numbers: JX070938-JX071040; JX071088-JX071779; JX071812-JX071899; JX071950-JX072081; JX072129-JX072677; JX072678-JX072726; JX072728-JX072811; JX072821-JX072834; JX072845-JX072916.

### Intra-subtype Recombination Analysis

Analysis in this study was performed using RDP3, a software package for statistical identification and characterization of recombination events in DNA sequences [30]. RDP3 simultaneously utilizes a range of non-parametric recombination detection methods: RDP, GENECONV [38] BOOTSCAN [39], [40], MAXICHI [41], [42], CHIMAERA [41], SISCAN [43] and 3SEQ [44]. RDP3 treats every sequence within the analyzed alignment as a potential recombinant and systematically screens sequence triplets/or quartets to identify viruses that contain a recombinant and two sequences that could serve as parents while performing a statistical evaluation of recombination signals [30]. Such an approach eliminates the need for reference sequences, which makes analysis of viral quasispecies from epidemiologically unlinked patients more practical [45].

In this study, 1795 HIV-1 *env* quasispecies from 45 subjects infected with HIV-1 subtypes A1, C, or D [19] were analyzed for evidence of intra-subtype recombination including subsets sampled at the early and the later time points over one year of follow-up. The default RDP3 parameter settings were used. The HIV-1 sequence was considered to be recombinant if the recombination signal was supported by at least three methods with p-value of ≤0.05 after Bonferroni correction for multiple comparisons implemented in RDP3 [30], [46].

The breakpoint position and recombinant sequence(s) inferred for every detected potential recombination event were manually checked and adjusted whenever necessary using recombination signal analysis implemented in RDP3.

### Determination of Recombination Patterns

The recombinant viral quasispecies were analyzed for recombination patterns based on the distribution of breakpoints across quasispecies. The Bootscan method implemented by RDP3 was used to identify the breakpoint position for each recombinant. The recombinants were considered to be *unique* if each recombinant variant was represented by a single copy. If more than one sequence showed evidence of similar recombination breakpoints, the recombinants were considered *enriched*.

### Comparison of HIV-1 Intra-subtype Recombination between HIV-1 Single and Multiple Variant Infections

HIV-1 single and multiple variants were reported in the recent study based on phylogenetic branching topology [19]. Comparison of HIV-1 intra-subtype recombination between the subjects infected with HIV-1 single and multiple variants was performed in order to assess whether the intra-subtype recombination differs between the two groups.

### Statistical Analysis

Descriptive statistics were quantified using SigmaPlot v. 7.0. Comparisons of continuous outcomes between two groups were based on the Mann-Whitney Rank Sum test. The bootstrap support values for the inferred phylogenetic trees were computed by MEGA 5.0. All reported p-values for RDP3 uses Bonferroni correction for multiple comparisons. Regression analysis was performed using linear regression and the spearman Rank test.

## Results

### Recombination Analysis

**Figure 1:**
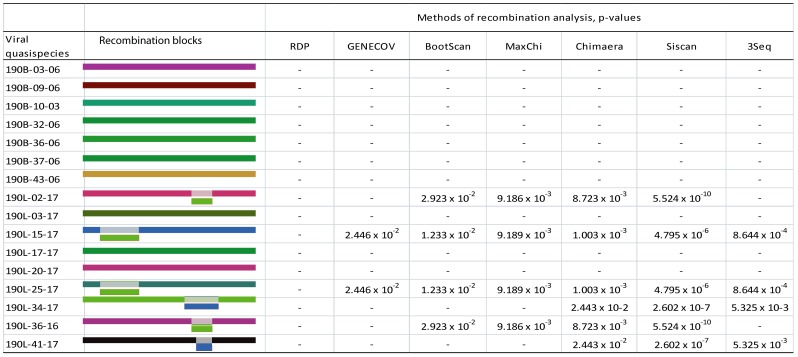
HIV-1 intra-subtype recombination analysis. RDP3 methods with supporting p-values and RDP3 recombination blocks are shown for subject 190 infected with six intra-subtype recombinant variants of HIV-1 subtype A1. Colored rectangles represent sequence fragments and the predicted recombinant regions are indicated by small boxes. Absence of p-value indicates no recombination event with the specified method. In the viral quasispecies column, “B” indicates early time point and “L” indicates later time point.

The HIV-1 *env* gp120 V1-C5 quasispecies from 45 subjects infected with HIV-1 subtypes A1, C, or D [19] were analyzed for evidence of intra-subtype recombination. The median *env* sequences analyzed per subject per two time points was 38 (IQR 28–50) over one year of HIV infection. Recombination analysis was performed using seven methods implemented in RDP3. The HIV-1 sequence was considered a recombinant if the recombination signal was supported by at least three methods with p-value of ≤0.05 after Bonferroni correction for multiple comparisons [30], [46]. Figure 1 shows an example of recombination analysis for subject 190 infected with six HIV-1 intra-subtype recombinant variants of subtype A1. Similar analyses were performed for all 45 subjects.

We found that 38 (84%) out of 45 subjects were infected with intra-subtype recombinant viruses. Among those, 22 subjects were infected with HIV-1 subtype A1, 13 subjects were infected with HIV-1 subtype C and 3 subjects were infected with HIV-1 subtype D (Table 1).

**Table 1:** HIV-1 intra-subtype recombinant viruses among female bar and hotel workers in Moshi, Kilimanjaro region, Tanzania, during 2004–2007.

HIV-1 subtypes	Subjects, n	Subjects harboring HIV-1 intra-subtype recombinants, n (%)
A1	27	22(81%)
C	15	13(86%)
D	3	3(100%)
Total	45	38 (84%)

The percentages of subjects with HIV-1 intra-subtype recombinants were calculated from analysis of the combined sets (early and later time points) of the viral quasispecies.

### Distribution of Recombination Breakpoints

**Figure 2:**
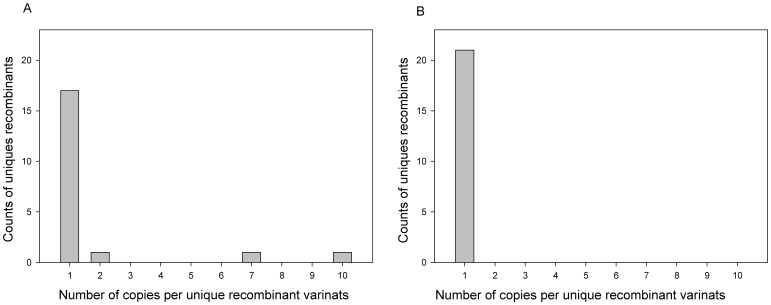
A: Distribution of unique intra-subtype recombinant variants in subject 168 infected with HIV-1 subtype A1. The number of unique recombinant variants was 20 (53%). The enriched recombinant variants were represented by 2, 7 and 10 copies. **B:** Distribution of unique intra-subtype recombinant variants in subject 107 infected with HIV-1 subtype A1. All 21 recombinant variants were unique.

The viral quasispecies from 38 subjects with evidence for intra-subtype recombination were analyzed for the distribution of recombination breakpoints. Presence of multiple unique recombination breakpoints among viral quasispecies in a particular subject suggests ongoing HIV-1 recombination processes. Based on the distribution of recombinant breakpoints we observed two types of recombination patterns: 1) all recombinants were unique without dominance of any particular variant, and 2) some recombinant variants were enriched. A unique distribution of breakpoints without dominance of any particular variants was found in 15 (40%) subjects. In contrast, in 23 (60%) subjects some recombinants were enriched, apparently suggesting their selective advantage. In all recombinants putative breakpoints along the gp120 appeared to be distributed across the V1-C5 region. Figure 2A shows that in subject 168, 20 (53%) of 38 recombinant variants of HIV-1 subtype A1 were unique, and three of those were enriched and were represented by 2, 7 and 10 copies. In contrast, Figure 2B shows subject 107 infected with 21 (100%) unique recombinants of HIV-1 subtype A1 without dominance of any particular variant.

Table 2 shows a summary analysis of the frequency of HIV-1 intra-subtype recombination and the frequency of unique intra-subtype recombinants.

**Table 2:** Summary analysis of frequency of intra-subtype recombination in 38 subjects from the combined sets (early and later time points) HIV-1 quasispecies.

Subject code	HIV-1 subtype	Total number of analyzed sequences	HIV-1 intra-subtype recombinants within the pool of viral quasispecies		Unique intra-subtype recombinants* within the pool of viral quasispecies	
			n	%	n	%
87	A	37	13	35%	12	92%
107	A	33	21	64%	21	100%
177	A	62	28	45%	28	100%
178	A	24	1	4%	1**	
190	A	16	6	38%	4	67%
355	A	41	5	12%	2	50%
404	A	30	1	3%	1**	
405	A	17	1	6%	1**	
620	A	38	23	61%	17	74%
697	A	32	5	16%	5	100%
740	A	18	7	39%	5	71%
807	A	41	1	2%	1**	
905	A	28	19	68%	19	100%
909	A	73	29	40%	23	79%
945	A	35	17	49%	16	94%
20	A	76	30	39%	21	70%
46	A	28	4	14%	2	50%
65	A	63	32	51%	29	91%
168	A	55	38	69%	20	53%
204	A	41	12	29%	10	83%
237	A	40	24	60%	18	75%
245	A	50	43	86%	21	49%
27	C	58	32	55%	30	94%
66	C	32	14	44%	10	71%
80	C	32	24	75%	16	67%
276	C	56	16	29%	9	56%
321	C	28	8	29%	8	100%
558	C	47	2	4%	2	100%
603	C	40	21	53%	17	81%
838	C	45	1	2%	1**	
968	C	48	28	58%	26	93%
171	C	25	1	4%	1**	
201	C	38	23	61%	23	100%
291	C	63	12	19%	11	92%
498	C	14	4	29%	4	100%
530	D	17	17	63%	15	88%
733	D	95	1	1%	1**	
871	D	13	4	31%	3	75%

The percentages (%) of unique intra-subtype recombinants were calculated from the HIV-1 intra- subtype recombinant variants.

* The proportion of unique recombinants within the identified HIV-1 intra- subtype recombinant variants.

** For the subject with a single intra-subtype recombinant variant, the proportion was not calculated.

### Analysis of Subsets: Sampling Over One Year of HIV-1 Infection

To assess whether patterns and frequency of intra-subtype recombination are associated with the time of sampling, we analyzed subsets of viral quasispecies sampled two time points over one year. At the early time point of sampling, 29 (69%) out of 42 subjects showed evidence of HIV-1 intra-subtype recombination, while at the later time point of sampling, 36 (82%) out of 44 subjects had recombinant viral quasispecies (Table 3). (Note that for subjects 321, 404, and 405, the HIV-1 quasispecies were available only at the later time point, while for subject 740 the HIV-1 quasispecies were available only at the early time point.) Although the proportion of subjects with evidence of intra-subtype recombination increased from 69% to 82% over one year, the difference was not statistically significant (p = 0.213, Fisher exact test).

**Table 3:** HIV-1 intra-subtype recombinant viruses among female bar and hotel workers over one year of follow-up.

Time point	Subjects, n	Subjects harboring intra-subtype recombinants, n (%)
Early	42	29 (69%)
Later	44	36 (82%)

45 subjects were analyzed. Note that three subjects (321, 404, and 405) at the early time point of sampling and one subject (740) at the later time point of sampling were excluded from the analysis due to the unavailability of viral quasispecies.

To assess the consistency of intra-subtype recombinants detection, we examined the presence of recombination signal in paired subsets of individual viral quasispecies collected over one year of HIV-1 infection. In 24 (56%) out of 41 subjects recombinants were detected at both time points. In four subjects (171, 178, 276, and 733) recombinants were detected at early but not at the late time point. In nine subjects recombinant variants were detected at later but not at the early time point.

### Mean Pairwise Genetic Distances and HIV-1 Intra-subtype Recombination

We tested the hypothesis that genetic diversity is associated with the number of identified recombinants. We found a statistically significant direct association between the mean pairwise genetic distances and the number of intra-subtype recombinants within all analyzed sets or subsets at early time point (r^2^ = 0.412; p = 0.007), later time point (r^2^ = 0.467; p = 0.002) and in the combined sets (r^2^ = 0.521; p = 0.001).

### HIV-1 Intra-subtype Recombination and HIV-1 Plasma Viral Load

We hypothesized that the frequency of HIV-1 intra-subtype recombinants is associated with the levels of HIV-1 RNA in plasma. We found a statistically significant direct association between HIV-1 intra-subtype recombination and HIV-1 viral load at the early time point (r^2^ = 0.379; p = 0.013) but not at the late time point (r^2^ = 0.215; p = 0.160). Also, the HIV-1 RNA load at the early time point was not associated with the number of recombinants at the late time point (r = 0.146; p = 0.344). It should be noted that HIV-1 RNA was undetectable in 10 subjects at the late time point, and no information regarding anti-retroviral treatment (ART) was available. We cannot exclude that at least some subjects started ART during the study.

### Comparison of HIV-1 Intra-subtype Recombination between HIV-1 Single and Multiple Variant Infections

In this study 38 out of 45 subjects showed evidence for HIV-1 intra-subtype recombination. Among the 38 subjects, 26 were infected with HIV-1 single (n = 26), while 12 subjects were infected with HIV-1 multiple variants [19]. The viral quasispecies of the subjects infected with the HIV-1 single and multiple variants from the recent study [19] were compared with respect to HIV-1 intra-subtype recombination. We found no statistically significant difference in recombination between the subjects infected with HIV-1 single (n = 26) or multiple variants (n = 12, p = 0.064). However, after excluding recombinant quasispecies, we found multiple HIV-1 variants in three subjects previously classified as infected with single HIV-1 variant (Table 4). This correction resulted in a statistically significant difference in recombination between the subjects infected with HIV-1 single (n = 23) and multiple variants (n = 15, p = 0.008).

**Table 4:** Neighbor-joining phylogenetic analysis bootstrap support values of HIV-1 quasispecies from 11 subjects classified as infected with HIV-1 single variant infections from a previous study [19].

Subject code	Number of quasispecies	Recombinant quasispecies	Quasispecies with recombinant variants		HIV-1 variant	Quasispecies without recombinant variants		HIV-1 variant
			Clusters (bootstrap values)			Clusters (bootstrap values)		
27	58	32	100%	<80%	1	100	100	2
107	33	21	<80%	<80%	1	98%	89%	2
178	24	1	99%	<80%	1	99%	<80%	1
321	28	8	99%	<80%	1	99%	<80%	1
404	30	1	100%	<80%	1	100%	<80%	1
558	47	2	<80%	<80%	1	<80%	89%	1
697	32	5	<80%	<80%	1	85%	99%	2
871	13	4	100%	<80%	1	<80%	100	1
909	73	29	<80%	<80%	1	<80%		1*
945	35	17	<80%	99%	1	<80%		1*
968	48	28	<80%	100%	1	<80%		1*

* One cluster of diversified variant (single variant).

Note: Bootstrap support values of ≥80% were considered to be significant.

## Discussion

This study aimed to determine the prevalence of HIV-1 intra-subtype recombination in a cohort of hotel and bar workers in Moshi, Tanzania. We found that HIV-1 *env* gp120 quasispecies in 38 (84%) out of 45 subjects showed evidence of intra-subtype recombination, suggesting that intra-subtype recombination is more common than previously thought. The frequency of intra-subtype recombination was higher than the frequency of inter-subtype recombination (8%) [19] in the same population, where HIV-1 multiple subtypes A, C, and D are circulating. These results are consistent with the recent studies among subjects infected with HIV-1 subtype C [18] and HIV-1 subtype B [47] viruses. Among subjects identified with pure HIV-1 subtypes and evidence of intra-subtype recombination, 22(81%) were infected with subtype A1, 13 (86%) with subtype C, and three (100%) with subtype D.

The high prevalence of intra-subtype recombinant viruses in this population may be due to multiple mechanisms. The relatively high frequency (27%) of HIV-1 infections with multiple viral variants of the same subtype [19] can be combined with the ability of HIV-1 reverse transcriptase enzyme to switch between the RNA templates during reverse transcription [48]. Furthermore, intra-subtype recombination might not necessarily require co- or super-infections, because as time of HIV infection goes by, the intra-host diversification produces a pool of viral quasispecies that can be used as distinct templates for intra-subtype recombination. Also, the association between genetic diversity and recombination in this study is consistent with the previously published data [47]. A high proportion of HIV-1 intra-subtype recombination in the V1-C5 region of the *env* gene may also be due to this region of gp120 encodes surface glycoproteins and can be under relatively high selective pressure from host immune system. The distribution of recombination breakpoints across the analyzed gp 120 region was similar to results reported in the recent study by Lamers et al [47]. A comparison of recombination patterns between *env* and other HIV-1 genes warrants further studies. The potential reasons for the relatively low prevalence of inter-subtype recombinant viruses reported in this population have been described previously [19].

The identified recombinants in this study were all unique, and none of the subjects shared recombination patterns/or breakpoints with other subjects, suggesting ongoing recombination processes on a population level in the local HIV/AIDS epidemic. Within the individual quasispecies, we observed selective enrichment of recombinant variants in 23 (60%) subjects, while in 15 (40%) subjects all recombinants were unique without dominance of any particular variant.

Analysis of the HIV-1 quasispecies over one year of sampling indicated that the proportion of subjects with HIV-1 intra-subtype recombination increased from 69% to 82%. Although the difference was not statistically significant, possibly due to small sample size, the increased number of subjects with recombinants at the later time point suggests evidence of ongoing recombination processes among the circulating viruses. In 56% of the subjects, recombinants persisted at both the early and the later time points over one year of infection, while in four subjects recombinants were detected only at the early time point of sampling. It is possible that recombinant variants were not detected due to low levels of replication, or substitution with non-recombinant viruses due to lower fitness. For the nine subjects in which recombinants were detected at the later time point only, it is also possible that the recombinants had a selective advantage of fitness. It has been shown that most recombinants do not survive long in the host, but a few persist throughout the infection, and some of the recombinants even displace the original infection indicating superior fitness and competitive ability [49].

Thus, a large proportion of female bar and hotel workers were infected with HIV-1 intra-subtype recombinant viruses regardless of whether the analysis was based on the early, later or combined sets.

We found a significant association between mean pairwise genetic distances and the number of HIV-1 intra-subtype recombinants. Our findings are consistent with previous report on gp120 in HIV-1 subtype B infection [47]. Also, we found significant association between HIV-1 intra-subtype recombination and the HIV-1 plasma viral load, which is consistent with previous studies [21], [50].

We compared HIV-1 intra-subtype recombination among subjects infected with single and multiple variants [19]. Without taking into account intra-subtype recombination, there was no statistically significant difference between the groups. However, after the adjustment for intra-subtype recombination, the difference became statistically significant. This was consistent with the previous reported studies of HIV-1 inter-subtype recombination and multiplicity of HIV-1 infection [50]–[52].

In the present study we have reported a high proportion of women in Tanzania infected with HIV-1 intra-subtype recombinant viruses. Recently we reported that 12 (27%) out of 45 subjects were infected with HIV-1 multiple variants of the same subtype, whereas the remaining 33 subjects were infected with HIV-1 single variants [19]. Furthermore, 11 out of 45 subjects were classified as infected with HIV-1 single variant infections although the viral quasispecies formed two phylogenetic clusters, the bootstrap support values were not significant enough to assign HIV-1 multiple variant infections (Table 4). To address whether intra-subtype recombination has an impact on phylogeny, the analysis was adjusted for intra-subtype recombination. After intra-subtype recombinant variants were excluded three more subjects from our recent study [19] were identified to be infected with multiple HIV-1 variants. Previous studies have also shown that recombination has an impact on phylogenetic inferences [18], [29], [53].

Although intra-subtype recombination has been reported previously [11], [14]–[18], [47], [54], [55], the present study has a larger sample size and reveals a higher prevalence rate, suggesting that intra-subtype recombination is more common than previously thought. To the best of our knowledge this is the first report of a high prevalence of intra-subtype recombination in the HIV/AIDS epidemic in Tanzania, a region where HIV-1 multiple subtypes A, C, D, and inter-subtype recombinant viruses co-circulate.

This study provides valuable information about the frequency of HIV-1 intra-subtype recombination in a selected study population in Tanzania. Results presented should be considered in view of the following limitations. First, the duration and stage of HIV-1 infection was unknown, and the study had no power to determine whether the HIV-1 intra-subtype recombination was due to co-infection, super-infection, or both. Second, in order to increase the effectiveness of RDP3 methods to detect recombinants, generation of at least 20 sequences per subject would have been ideal however, some of the subjects had a low number of quasispecies. Third, some of the subjects had undetectable plasma HIV-1 viral RNA, which is likely to be associated with efficiency of PCR amplification. Fourth, the current approach for identification of HIV-1 sequences with intra-subtype recombination has some limitations due to Bonferroni correction, which made the method sensitive to the number of analyzed quasispecies in some cases (four subjects in our study). However, we found no correlation between the number of analyzed sequences and the number of identified recombinants (data not shown). We suggest that the RDP3 method should be further validated and criteria for robust identification of HIV intra-subtype recombinants be developed.

In summary, this study demonstrated that a large proportion of female bar and hotel workers in Moshi, Tanzania are infected with HIV-1 intra-subtype recombinants. This is the first report of a high prevalence of intra-subtype recombination in the HIV-1/AIDS epidemic in Tanzania, a region where HIV-1 multiple subtypes co-circulate. HIV-1 intra-subtype recombination might increase viral diversity posing additional challenges to treatment and HIV-1 vaccine design.
